# Eating in the Amazon: Nutritional Status of the Riverine Populations and Possible Nudge Interventions

**DOI:** 10.3390/foods10051015

**Published:** 2021-05-06

**Authors:** Camila Lorena Rodrigues Machado, Maria Elena Crespo-Lopez, Marcus Augusto-Oliveira, Gabriela de Paula Arrifano, Barbarella de Matos Macchi, Amanda Lopes-Araújo, Letícia Santos-Sacramento, José Rogério Souza-Monteiro, Jacqueline Isaura Alvarez-Leite, Carlos Barbosa Alves de Souza

**Affiliations:** 1 Laboratório de Farmacologia Molecular, Instituto de Ciências Biológicas, Universidade Federal do Pará, 66075-110 Belém, PA, Brazil; camilalorenarodriguesmachado@gmail.com (C.L.R.M.); marcusoliveira@globo.com (M.A.-O.); arrifanogabriela@gmail.com (G.d.P.A.); amanda.lopes1647@gmail.com (A.L.-A.); letisacramentolfm@gmail.com (L.S.-S.); 2 Laboratório de Neuroquímica Molecular e Celular, Instituto de Ciências Biológicas, Universidade Federal do Pará, 66075-110 Belém, PA, Brazil; barbarella@ufpa.br; 3 Faculdade de Medicina, Campus de Altamira, Universidade Federal do Pará, 68372-040 Altamira, PA, Brazil; rogerio.souza.monteiro@gmail.com; 4 Departamento de Bioquímica e Imunologia, Universidade Federal de Minas Gerais, 313270-901 Belo Horizonte, MG, Brazil; jacque.alvarez.leite@gmail.com; 5 Núcleo de Teoria e Pesquisa do Comportamento, Instituto Nacional de Ciência e Tecnologia Sobre Comportamento, Cognição e Ensino, Universidade Federal do Pará, 66075-110 Belém, PA, Brazil

**Keywords:** Amazonian, riverine population, nutrition, anthropometric data, vulnerable, nudge interventions, behavior analysis

## Abstract

The Amazon is the largest tropical forest in the world and a source of healthy food, such as fruits and fish. Surprisingly, the Amazonian riverine population present an increased prevalence (as high as 58%) of non-communicable diseases, such as hypertension and insulin resistance, even higher than that described for the urban population of the Amazon. Therefore, this work aimed to analyze the nutritional status and associated risk of the riverine population. Body mass index, waist circumference (WC), waist-to-hip ratio, and neck circumference (NC) were evaluated, and risk analysis was assayed. Furthermore, data about occupation and the prevalence of consumers of the different groups of food were analyzed. All anthropometric parameters revealed high proportions of individuals at risk, WC and NC being the factors that had more high-risk women and men, respectively. Our data confirmed the characteristic profile of the riverine communities with a high number of fish consumers, but also observed different patterns probably associated to a phenomenon of nutrition transition. Based on our data, some nudge interventions that take into account the principles of behavior analysis are discussed and proposed for these populations, aiming to improve the nutritional status and avoid the long-term consequences of the results showed by this work.

## 1. Introduction

The largest tropical forest in the world is found in the Amazon River basin, most of it belonging to Brazil. The Brazilian Amazon (also known as Legal Amazonia in Brazil) includes more than 5 million square kilometers (about half of the USA territory) in nine states, where more than 17 million people live [1]. Despite the abundant natural resources in the region, some of the lowest Human Development Index scores in Brazil can be found there [2]. 

Although the natural environment provides healthy food, such as fruits and fish, many areas of the Amazon are contaminated with pollutants (e.g., due to the absence of a sewage system or the use of mercury in traditional gold mining) influencing the quality of water and food [3]. For instance, mercury contamination was already associated in these populations to neurological impairments and an increased risk of hypertension [4,5,6]. Furthermore, the arrival of electricity and the improvement of infrastructure in some regions may be contributing to a nutrition transition in some communities [7,8], because the presence of electricity allows the improved conservation of food and increases the commerce of different products. Nutrition transition is the shift in dietary consumption and energy expenditure that coincides with economic, demographic, and epidemiological changes. The resulting major shifts in diet are toward increased refined carbohydrates, sweeteners, edible oils, and other animal-source foods, as well as reduced legumes, vegetables, and fruits in the diet of traditional communities. Low- and middle-income countries face a rapid change in the nutrition transition toward increases in non-communicable diseases (NCDs) [9]. Dietary factors could be especially influencing the cardiovascular risk of Amazonian populations, since a recent report demonstrated a high prevalence (as high as 58%) of NCDs, such as hypertension and diabetes mellitus (DM) [10]. Interestingly, this prevalence was higher than that described for the urban population of the Amazon [10], making the evaluation of the nutritional status of the riverine population and the collection of dietary information urgent, which can support the design of tailored behavioral interventions for improving the nutritional status of this population. 

Nutritional assessment involves anthropometric data, enabling the identification of likely effects on health and nutrition [11]. The evaluation of nutritional status uses simple equipment, such as scales and measuring tapes, which are especially adequate for the monitoring of remote/isolated populations living in locations with precarious conditions. Although the use of diverse anthropometric parameters is recommended for an accurate evaluation, most of the studies performed on Amazonian riverine populations focused on body mass index (BMI) or waist circumference (WC) only. 

Therefore, this work aimed to analyze the nutritional status and associated risk in the Amazonian riverine populations. BMI, WC, waist-to-hip ratio (WHR), and neck circumference (NC) were evaluated, and a risk analysis (RA) was performed that integrated these parameters. Additionally, data about occupation and the prevalence of consumers of the different food groups were also analyzed and discussed. Based on these data, some nudge interventions [12], which take into account the principles of behavior analysis [13,14], are discussed and proposed, aiming to improve the nutritional status of these populations. 

Nudges are “any aspect of the choice architecture that alters people’s behavior in a predictable way without forbidding any options or significantly changing their economic incentives” [12] (p. 6). Choice architecture is the setting in which individuals behave, and comprises, for example, how default options are presented when there are many alternative choices, how information is afforded, and the order in which the options are presented. Nudges have been used when trying to promote behavioral changes related to health, dietary behavior, physical activity, saving and financial decisions, education, energy efficiency, and work performance, among other subjects (e.g., [15,16,17,18,19,20,21]). 

Although usually effective, the efficiency of the nudges can be improved [22]. One of the factors that can affect nudge efficiency as strategies to modify behaviors is the fact that nudges emphasize the antecedent aspects of the behavior control and rarely consider its consequences. For example, when pursuing to promote (1) healthier eating habits in students at a school cafeteria, healthier foods are placed in the most easily accessible places, or (2) to encourage the carrying out of school-related activities at home, text messages with reminders about the activities are sent to parents. However, these changes in the choice architecture could be more efficient if they also contemplated some consequences closer to the occurrences of the behaviors that are intended to be modified, as is usual in behavior analysis [23]. Behavior analysis emphasizes that behaviors are selected by their consequences [13,14]: those that increase the probability of a behavior occurring (reinforcers) and those that decrease this probability (punishers). Therefore, in addition to the main objective of this work, we discussed a simple set of nudges that incorporate the consequences of behaviors, which, when implemented, can encourage behavioral changes in the eating habits and physical activity in the Amazonian riverine populations included in this study.

## 2. Materials and Methods

### 2.1. Amazonian Riverine Population

**Figure 1:**
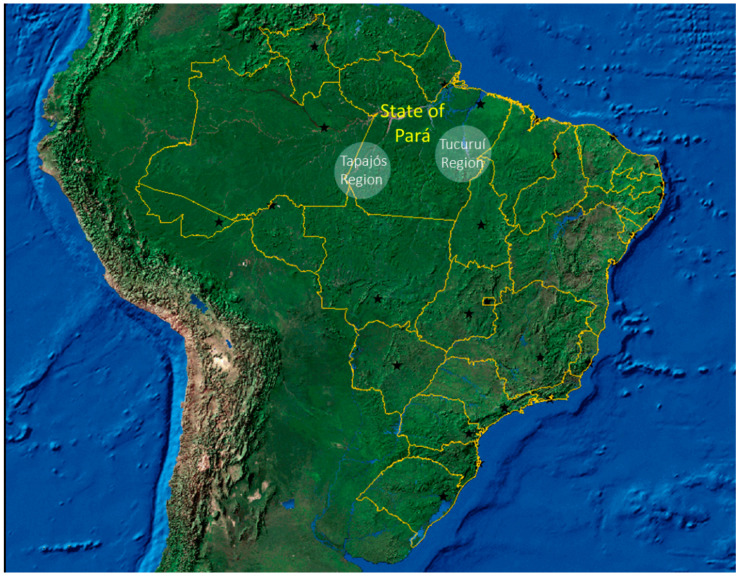
Map of Brazil showing the states (yellow lines) and their capitals (black stars). The two regions included in this study are indicated in white. Adapted from maps available at https://www.ibge.gov.br/geociencias/cartas-e-mapas/mapas-de-referencia.html (accessed on 12 February 2021).

In this study, riverine communities in two regions of the Amazonian basin were included: the Tapajós River (latitude 04°16′34″ S and longitude 55°59′01″ W) and the Tucuruí Lake (latitude of 3°46′10″ S and longitude of 49°40′27″ W) (Figure 1). Inhabitants of these communities live in small riverine villages or in widespread family-based houses on the main riverbanks. The river is the key element in the lives of these communities (used for transportation, commerce, hygiene, source of water and food, etc.). The Amazonian riverine population presents a specific profile with artisanal fishing and subsistence agriculture, both characterizing the basis of the economy of these communities [3]. Fish is usually the main protein source in the diet, with a large number of meals per week (seven or more), frequently contaminated with mercury [3,4,5,24]. Human exposure to mercury occurs in the two regions due to the influence of artisanal small-scale gold mining or large-scale projects, such as dams [25,26].

This project was publicized via radio, meetings, and direct communications with healthcare agents. Samples were collected from volunteer participants at community meeting places (e.g., local schools). Inclusion criteria were adults (≥18 and <75 years-old), both genders, and residents in the communities for more than two years (riverine residents). Exclusion criteria comprised drug dependence and patients with severe and chronic diseases or who had been on medication for the past two months prior to the study to avoid possible influences or restrictions in food intake. All of the participants were informed about the study and gave written consent. All of the procedures were approved by the National Council for Ethics in Research with Human Beings (CONEP, Brazil; CAAE #43927115.4.000.0018).

### 2.2. Identification and Prevalence of Consumers

Each participant was identified by a code to preserve their identity. Data, such as gender, occupation, and age, among others, were registered. The prevalence of consumers of the different food groups consumed by riverine populations (fish, meat, dairy products, eggs, breads and cereals, vegetables, flour, beans, and fruits) was analyzed using a qualitative questionnaire (seven-day recall). Responses to the questions (“do you eat X frequently, three or more times a week?”) were registered as positive or negative, and the frequency of positive responses was calculated for each food group, characterizing the prevalence of consumers. Processed food (commercially prepared food, often through processing, to optimize the ease of consumption and make it more attractive to the consumer) was identified as an observational recording, but it was not included in the questionnaire as a food group.

### 2.3. Anthropometric Evaluation

Weight, height, and waist, hip, and neck circumferences of each participant were registered without shoes, coats, jackets, caps, or any other accessory that could influence the final measurements. For weighting, the individual was positioned in the center of the digital scale (Omron, São Paulo, SP, Brazil) in an upright position, with arms extended along the body. The height measurement was obtained using a non-elastic measuring tape (Sanny, São Paulo, SP, Brazil) duly fixed on the wall of the collection site and a metal ruler fixed on the head perpendicularly to the wall. The participant was instructed to stay upright, with their weight distributed over both feet, the head positioned in the Frankfurt horizontal plane, arms extended along the body with the palms facing the thighs, heels touching the base, and the knees, shoulders, and buttocks touching the vertical surface (the reading was recorded with an accuracy close to 0.1 cm). The BMI was calculated as weight/(height)^2^, and each participant was classified according to it (Table 1).

The WC and hip circumference (HP) were measured with the participant standing in an upright position, using a non-elastic tape 2 m long (Sanny, São Paulo, SP, Brazil), with a precision of 0.1 cm [28]. The tape was snugly positioned at the horizontal plane midway between the lowest ribs and the iliac crest for the WC and at the widest portion of the buttocks for the HP [30,31,32,33]. The waist-to-hip ratio (WHR) was calculated as WC/HP and used to identify individuals at risk of cardio-metabolic disturbances related to obesity (Table 1). However, in the last few years, several studies have shown that the WC alone is more strongly associated with visceral fat than the WHR (reviewed [34]), so the participants were also classified according to WC values of risk (Table 1). The NC was measured at a point just below the larynx (thyroid cartilage) and perpendicular to the long axis of the neck, with the individual standing erect and the head positioned in the Frankfurt horizontal plane, with the gaze facing forward. The NC is a more recent method (compared to the WC and WHR) for differentiating fat distribution, as a marker of upper body subcutaneous adipose tissue distribution [29]. All of these measurements were used to identify possible cardio-metabolic risks according to Table 1.

Additionally, the data were used in a unified risk analysis (RA) to include the information of all parameters (BMI, WC, WHR, and NC) at the same time: individuals with no altered parameter or only one altered parameter were considered as no risk, and those with two or more altered parameters were considered as at risk. This simple analysis takes advantage of using more information than each parameter alone.

### 2.4. Statistical Analysis

The normality and homoscedasticity of the data were evaluated using the D’Agostino–Pearson test. Accordingly, the Student’s *t*-test or Mann–Whitney test was used to compare two means or medians, respectively. The chi-squared or Fisher’s test was performed to identify differences between prevalence. For all analyses, a *p*-value below 0.05 was considered significant.

## 3. Results

A total of 298 adults participated. After applying the inclusion/exclusion criteria, 234 individuals (143 from Tapajós and 91 from Tucuruí) were included, 67% being women. Table 2 shows the general characteristics of the individuals participating in this study. The number of women was about twice the number of men. The BMI and WC presented median/mean values indicative of cardio-metabolic risk. No difference in the gender distribution or mean/median values was detected between the two regions, except for the age (Table 2). Although participants of Tucuruí presented a median age older than those of Tapajós, no difference was detected between the regions in the distribution of participants according to age ranges (18–30 years, 31–43 years, 44–55 years, and 55–64 years) (chi-squared test, χ^2^ = 3.735, df = 3, *p* = 0.2915). Moreover, 55% of men were aged 44–55 years, a higher percentage when compared to women (30%). 

**Figure 2:**
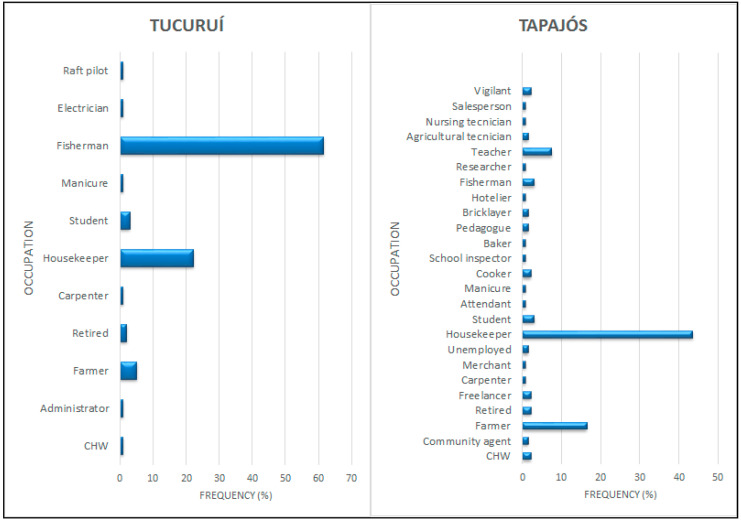
Occupations reported by the participants from the two Amazonian populations (Tapajós and Tucuruí). CHW = community health worker.

Fisherman was the most frequent self-reported occupation in the Tucuruí region, while housewife and farmer were the most prevalent in the Tapajós region for women and men, respectively (Figure 2).

According to BMI, about 60% of the participants were classified as pre-obesity/obesity (pre-obesity and obesity I, II, and III), followed by normal weight and underweight (Table 3). 

Analyzing different degrees of obesity, most of the individuals were considered pre-obese, followed by obesity degree I, with a higher prevalence in men (Table 4).

**Figure 3:**
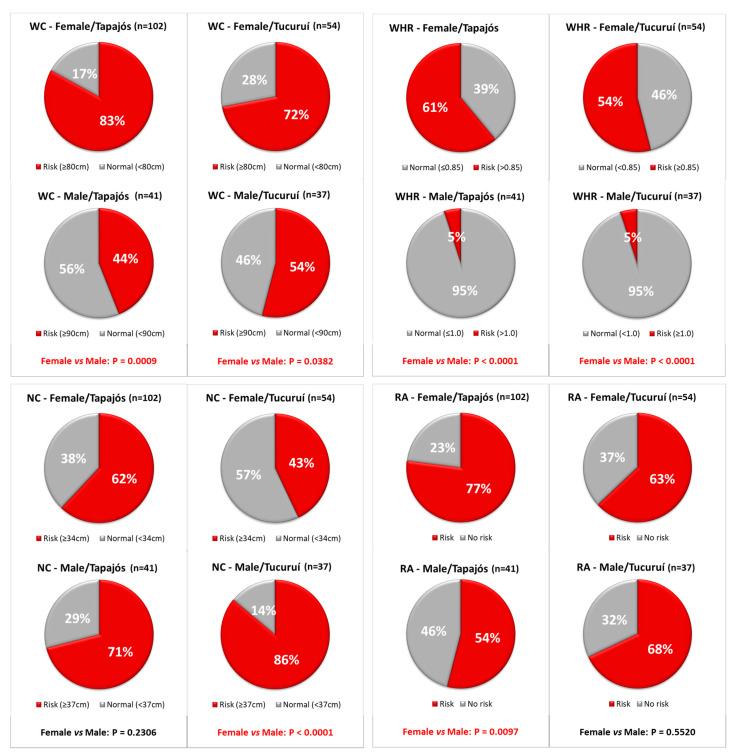
The prevalence of individuals at risk of cardio-metabolic impairment associated with obesity, according to the waist circumference (WC, above left), waist-to-hip ratio (WHR, above right), neck circumference (NC, below left), and risk analysis (RA, below right). Differences between genders (female vs. male) were evaluated by Fisher’s test for each region and highlighted in red when they were significant.

The WC and BMI averaged above the recommended limit (Table 1 and Table 2). When the WC was used to indicate visceral fat, no difference was detected in the prevalence of individuals at risk between the regions (Fisher’s exact test, *p* = 0.1168), as well as in median values (Table 2), but females had a significantly higher risk of developing obesity-related diseases than men in both regions (Figure 3).

As with the WC data, the WHR data were also measured as an indicator of visceral fat: approximately 40% of the participants were at risk of developing obesity-related diseases. According to the WHR, there was no difference in the prevalence of individuals at risk between the regions (Fisher’s test, *p* = 0.6622), as well as in median levels (Table 2), but a significantly higher risk for women compared to men was detected in both regions (Figure 3).

When the NC was evaluated as a marker of upper body subcutaneous adipose tissue distribution, 62% of the participants were at risk of obesity-related diseases, with no difference in the prevalence and median levels between the regions (Fisher’s test, *p* = 0.6622 and Table 2). Interestingly, the NC data showed that men were more vulnerable than women in the Tucuruí region (Figure 3).

The RA considers without risk individuals with no altered parameter or only one altered parameter, and individuals at risk as those with two or more altered parameters. Accordingly, 69% of the participants showed a risk of developing obesity-related diseases, with no differences between the regions (Fisher’s test; *p* = 0.3611). Interestingly, the RA showed that, in the Tapajós region, the prevalence of women at risk was higher than that of men, but no difference between women and men was detected in the Tucuruí region (Figure 3).

**Figure 4:**
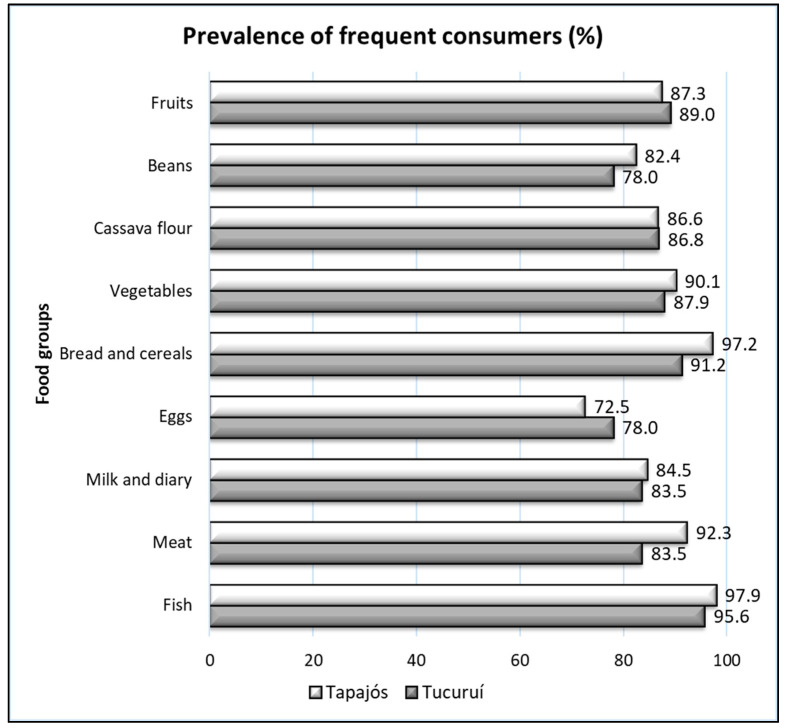
The prevalence of consumers (three or more times a week) of different food groups in the Tapajós (n = 143) and Tucuruí (n = 91) regions.

When analyzing self-reported feeding, it was observed that the intake of fish and cereals was widespread in the entire population (Figure 4), confirming the profile of fish consumers of the riverine populations. Furthermore, a large number of participants consumed fruits and cassava flour in both regions, Tapajós and Tucuruí. A lower prevalence of consumers was found for eggs. The large difference in the profile of the two regions corresponded to meat intake, and the Tapajós region showed a higher proportion of consumers (Figure 4).

**Figure 5:**
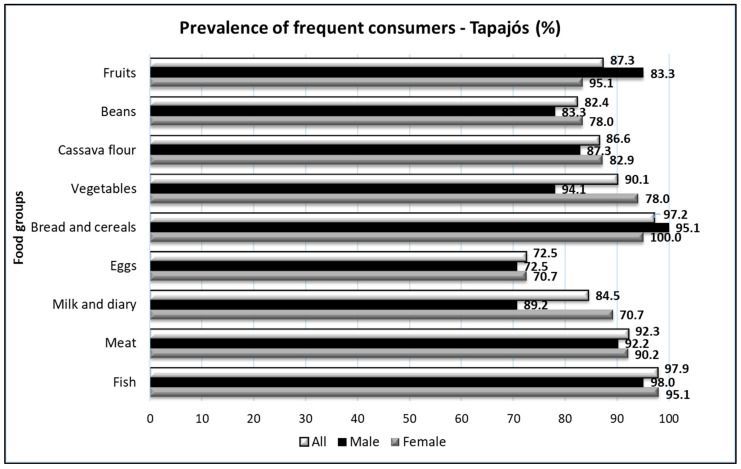
The prevalence of consumers (three or more times a week) of different food groups in all participants from the Tapajós region (All, n = 143) according to gender, male (n = 41) and female (n = 102).

**Figure 6:**
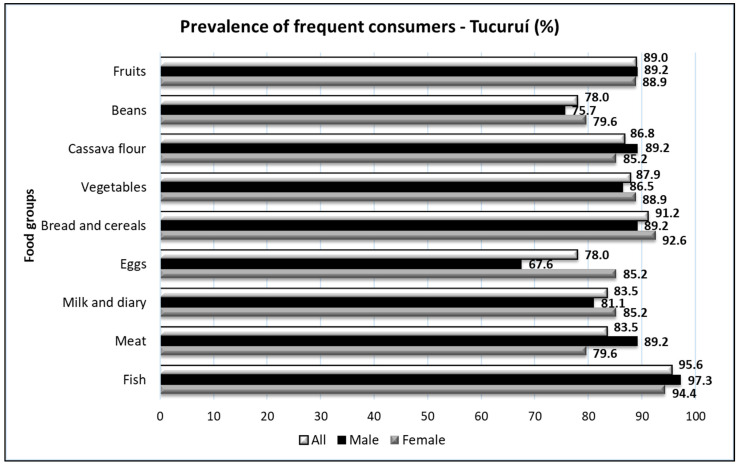
The prevalence of consumers (three or more times a week) of different food groups in all participants from the Tucuruí region (All, n = 91) according to gender, male (n = 37) and female (n = 54).

Interestingly, when responses of women and men were analyzed separately, we obtained additional information. In the Tapajós region, large differences (ten or more percentage points) were found in the intake of fruits (lower prevalence of consumers in women) and vegetables (lower prevalence in men) (Figure 5). In the Tucuruí region, a significantly lower percentage of men consumed eggs when compared with women (Figure 6). Furthermore, a lower percentage of women reported eating meat regularly (Figure 6). 

**Figure 7:**
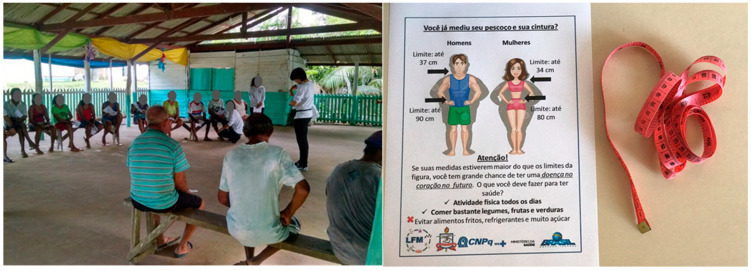
Reporting data to the participants (left). In addition to the individual results and public lectures, all participants of this study received a kit with a tape and an informative folder with limits of the anthropometric measurements.

After data collection and analysis, an individual clinical report was delivered to each participant with data of anthropometry and eating habits, among other data. In addition to the individual report, a kit with a measuring tape and a folder explaining how to measure the neck, waist, and hip circumference, the anthropometric limits, and the health problems associated with exceeding these limits was delivered to each participant (Figure 7). Furthermore, public lectures were performed in each location to inform about the conclusions regarding the epidemiological data.

## 4. Discussion

This study found that Amazonian riverine populations have a traditional profile, with fishing and farming as the main occupations (Figure 2). Despite this contact with nature, nutritional status, evaluated with four anthropometric parameters (BMI, WC, WHR, and NC) and a unified analysis of risk (RA), revealed alarming values and prevalence of individuals at risk of cardio-metabolic disturbances (Table 2, Table 3 and Table 4 and Figure 3). BMI and WC averaged values above the recommended limits (Table 1 and Table 2). Moreover, the highest prevalence was detected with the WR and NC for women and men, respectively (Figure 3). A high number of fish consumers was confirmed, but the number of meat consumers in Tapajós was relatively higher than in Tucuruí (Figure 4 and Figure 5). A simple kit for anthropometric monitoring (Figure 7) was delivered to each participant, but additional tailored behavioral interventions can be proposed based on our data.

Epidemiological studies concerning the nutritional status of the Amazonian populations are scarce (e.g., [8]), probably due to, among other causes, the remote/isolated location of these communities. Although a prolonged period of data collection can increase the number of participants in a study, this often becomes logistically impracticable, since the large geographical displacement and precarious infrastructure of these communities make it very difficult to stay for a long time. In this context, our final sample size (234 participants) is similar or superior to the number of participants that can be found in the few epidemiological studies performed on these populations (e.g., [7,8]). Moreover, although the literature demonstrated the usefulness of the parameters used in this study for analyzing nutritional status [32], to the best of our knowledge, this is the first time that all of these parameters were applied to an analysis of the Amazonian population. 

A higher proportion of women participated in our study, especially in the Tapajós region (71% of all participants) (Table 2). This is frequent in epidemiological studies of Amazonian populations, and it has been attributed to a higher concern about health [10], with women self-reporting more symptoms of illness [33]. Our data about occupation, showing a high frequency of housekeepers in both regions (Figure 2), confirm previous descriptions of Amazonian populations observing that women were responsible for all domestic work, in addition to child care [7]. Furthermore, fishing is widely practiced in these small communities that depend on natural and local resources [35]. This is confirmed by the high prevalence of fishermen in the Tucuruí region (Figure 2). In the Tapajós region, farming was frequent as an occupation, pointing to the traditional agriculture also practiced by riverine people. Food production (especially cassava) has always been present in the Amazon [36]. This knowledge about the occupational profile is relevant, since frequently in these populations, their occupation is the only physical activity practiced by these individuals. As previously reported, the little knowledge of riverine people about the importance of physical activity, in most cases, means that the only activities practiced are occupation-related [37], thus interfering with anthropometric data and the definition of biotype.

The application of anthropometric measures for diagnosis, such as those performed in the present study, is especially interesting in the evaluation of isolated populations, such as the Amazonian riverine communities, due to the fact that it presents an easy, non-invasive, low-cost technique and is widely applicable [32]. According to BMI, the median values were above the recommended limits (Table 1 and Table 2). Moreover, in this study, more than half of the population was at risk for obesity (pre-obesity) or obese (obesity I, II, and III), with a prevalence of 57% and 56% for Tapajós and Tucuruí, respectively (Table 3 and Table 4). The absence of significant differences between the regions strengthens the idea that the regions have similar risk profiles, being quite representative of the Amazonian riverine communities. Furthermore, these results are very worrying, since they are above those reported by the World Health Organization (WHO) [38]: 39% of adults are already overweight worldwide. Our findings become even more worrying when considering that participants were selected using the inclusion and exclusion criteria to observe a relatively healthy population, in which high rates of obesity would not be expected. Surprisingly, this prevalence in the riverine population is also above that described for urban populations in the Amazon: for example, in 2019, the capital of the state of Pará, Belém, showed a prevalence of 53.3% overweightness and 19.6% obesity [39]. This fact is worrying and relatively unexpected, as the inhabitants of urban areas are closer to industrialized foods, while residents of rural areas are closer to natural foods. Some factors could be contributing to this phenomenon of high BMI, such as the reduced physical activity practiced by the riverine population [7] or a slight increase in purchasing power due to economic benefits from government income transfer programs (such as Bolsa Família, a benefit for keeping children at school) that may allow them to buy junk food, replacing traditional practices, such as agriculture, and leading to the nutrition transition [8]. In the communities of the Tapajós region, it was possible to observe small markets with industrialized food, such as soft drinks, cookies, and chocolates. However, in the Tucuruí region, the riverine inhabitants of the islands often did not have the money to buy certain foods, given that the expense involved not only the purchase of food, but displacement to the main city by canoes (no markets are present in the islands). Therefore, our data support the necessity of conducting a more in-depth study in Tucuruí to discern the real causes of the high prevalence of obesity found there.

To improve the detection of individuals at risk, in our study, additional anthropometric parameters were used, such as the WC (an indicator of abdominal fat and excess visceral fat and related to metabolic changes), that also present mean values above the recommended limits (Table 1 and Table 2). Information about the WC helps to prevent or detect morbidity and cardio-metabolic problems [32,40]. Interestingly, this parameter detected more individuals at risk than the BMI, indicating a higher prevalence of risk (72–77%), especially for women (as high as 83%) (Table 3 and Figure 3). The WC is highly influenced by the fat located in the abdomen, revealing abdominal obesity. Although this type of obesity is more frequent in men [40], in this study, we found that it is more prevalent in women, probably contributing to an increased cardiovascular risk among women of the Amazonian riverine populations. Among the possible causes, occupation could be partially responsible for this result. In the Tapajós region, women mainly do housework, which requires less energy expenditure than farming (the main occupation among men). These characteristics are inserted in a cultural context that was sustained for many years and appointed the man as the one responsible for practicing activities carried out outside of the house and require greater physical strength and energy cost [41]. Furthermore, it is important to highlight that body fat distribution has a strong genetic component [42]. The Amazonian riverine populations are a mixture of African, European, and indigenous origins [26], and the distribution of fat could be influenced by several factors, such as ethnicity and gender [43]. In this context, the WC provides important information, in addition to the BMI, to predict health risks related to obesity, increasing the quantity of information obtained when compared to the use of the BMI alone. The BMI does not distinguish between the weight associated with muscle or body fat and, therefore, it is important to evaluate body composition using other parameters. Moreover, we used the cutoff point of the WC for South American populations (Table 1), proposed by the International Diabetes Federation [27], to guarantee reliable results. Our results demonstrated for the first time that the WC is the parameter that detected more individuals at risk related to nutritional status and cardiovascular risk in women in the Amazonian population. 

The WHR is considered as an indicator to verify the type of body fat and the metabolic disorders related to visceral fat [44]. In our study, the WHR was especially useful to monitor the nutritional status of women in Amazonian populations, detecting 54–61% of women at risk (Figure 3). However, the WHR was the parameter that detected the lowest amount of men at risk (only 5%), below the risk detected by all of the other parameters, including the BMI, and with a significantly different response than that of women. This different sensitivity of the WHR according to gender could be partially related to the differences in fat accumulation in the android and gynoid forms [11], but the influence of additional factors, such as genetic background due to the trihybrid origin, cannot be discarded. Studies reporting the fat distribution or nutritional status of the riverine population in the Amazon are extremely scarce, and this absence of data would eventually avoid the need to establish customized cutoff points for these populations by international and national agencies. Our work contributes to revealing particular characteristics of the nutritional status and biotype of the Amazonian populations, and our alarming data support the need for further studies in these populations. 

Interestingly, the NC was the parameter that detected more men at risk related to nutritional status and cardiovascular risk (Figure 3). The NC is strongly associated with metabolic parameters and the thickness of epicardial fat [45]. In the upper part of the body, we can find epicardial fat, which is the visceral fat that develops vascular protective function in normal physiological conditions. It is a source of several anti-inflammatory and anti-atherogenic cytokines, which have a protective role against circulating fatty acids at high levels and energy when there is a high demand [46]. However, when epicardial fat is extensive, it can play a significant role in the development of cardiovascular and other metabolic problems by modulating the activity of the coronary artery and the pathogenesis of coronary artery disease [47]. The NC is a good anthropometric indicator, because it identifies the accumulation of fat in the upper body, not influenced by respiratory movements or postprandial abdominal distention [48]. The NC has been largely associated with the development of cardiovascular problems, such as metabolic syndrome, coronary artery disease, hypertension and diabetes mellitus [29,49]. In this work, the NC revealed that more than 60% of the participants were at risk for possible metabolic complications in both regions, and that the prevalence of men with a risk related to these complications is higher than that of women (Figure 3). These data would explain the previous findings of the significantly higher median blood pressure and hypertension prevalence in men compared to women [10]. However, additional studies are needed to better understand the higher prevalence of women at risk showed by the other anthropometric parameters.

The use of different anthropometric parameters in this work unprecedentedly allowed the performance of a robust evaluation of nutritional status. The RA included all of the strengths of each parameter, making the analysis more robust and reliable. Individuals at risk detected by the RA presented two or more altered anthropometric parameters, taking into account the results of the BMI, WC, WHR, and NC. With this analysis, it was possible to detect 65–72% of individuals at risk, with no significant difference between the regions in the total prevalence. However, when analyzing by gender, the female population in the Tapajós region had a significantly higher percentage of individuals at risk than that of men (Figure 3). In contrast, in the Tucuruí region, the prevalence of individuals at risk, although also high, does not differ between men and women. Thus, in the Tapajós region, there seems to be a different factor that affects women especially, although this disturbance does not seem to be enough to affect the prevalence of the total population compared to other Amazonian communities. Factors such as age do not seem to explain this difference between men and women found in Tapajós. In our study, the young adults predominated among women (18–43 years old), and this would contribute to a lower risk. Based on our preliminary results and previous studies, a possible hypothesis is that the different main occupation of women (housewives in Tapajós and fishing in Tucuruí) and the more facilitated access to junk food in Tapajós would be responsible, at least in part, for the higher risk found in women. Additional studies are urgently necessary to confirm this hypothesis and for a better understanding of the nutrition transition phenomenon in the Amazon. Additionally, our work demonstrated the importance of using two or more anthropometric parameters to assess nutritional status in these studies. The analysis of an individual parameter may provide non-conclusive data, compromising the final diagnosis.

As a preliminary approach, we also evaluated the number of participants that consumed different food groups. This kind of evaluation is especially challenging in remote/isolated populations, such as those included in the present work, due to high limitations, such as monitoring the exact estimate of the amount of eaten food or the difficulty of the interviewees to understand the questions (some of them do not write or read), among others. However, our preliminary results point to interesting facts. 

First, the high number of fish consumers in these populations was confirmed (Figure 4). Previous studies described a mean individual intake of seven or more meals of 141 g of fish per week [24]. Although fish is a known source of ω-3 and ω-6 fatty acids, elements with a cardio-protective function [50], in the Amazon, it is frequently contaminated with mercury, especially the piscivorous species [5,26,51]. In both regions, the riverine inhabitants reported consuming piscivorous fish, such as “tucunaré” (peacock bass, Cichla spp.) or “dourada” (gilded catfish, *Brachyplatystoma flavicans*), several times a week [24,25]. Human exposure to this metal has been associated with cardiovascular changes [52,53], and a high prevalence of NCDs, such as hypertension and DM, were recently described in these populations [10], making it even more important to monitor the nutritional status of these populations when this environmental factor is present. 

Second, a higher number of fruits, cereal, and cassava flour consumers was also detected (Figure 4). These are traditional and accessible foods in the diet of Amazonian populations. The numerous consumers of fruits are a positive fact, since, in the urban populations of Brazil, especially communities with a low income, fruit intake is lower than recommended (400 g/day) [54]. Amazonian fruits, such as açaí (*Euterpe oleracea*), are endemic, widespread, and with a high potential to protect organs, such as the heart or brain, even against the deleterious effects of mercury [55,56,57,58]. Furthermore, the lower number of egg consumers is also good news, considering the high content of cholesterol of this product, with the prevalence of hypertension already observed in these populations [10] and the levels of individuals at risk detected in our work.

A very traditional food in these populations is cassava flour, described as a dominant food and providing 50% of the total energy in the diet and 65% of the total carbohydrates [7,8]. In the interviews, participants frequently reported consuming 3–6 tablespoons of this flour per meal. Although the prevalence of consumers of this food was similar in the two regions, there could be differences in access. The traditional cassava cultivation practiced by these populations involves energy expenditure, but this practice has decreased in the Tapajós region, with easier access to this flour already processed in the markets [7]. Additional studies could analyze whether this difference could be contributing to a different energy expenditure in the two regions. Furthermore, bread and rice were the main cereals consumed by most participants (bread was consumed mainly for breakfast, between 100–200 g, and rice was consumed mainly for lunch and dinner, with the consumption of five or more spoons). This high intake of carbohydrates could be contributing to the worrying nutritional status detected in this work.

The higher difference between the two regions was the prevalence of meat consumers. According to the WHO [38], beef is a part of the eating habits of Brazilians. Passos et al. [24] brought attention to the partial substitution of fish for beef and chicken in the Tapajós region. Considering that fish intake is associated with human exposure to mercury, the partial substitution of fish for other meat in the Tapajós River basin, but not in Tucuruí Lake, could explain the significant differences of mercury exposure between the two regions [5,25], but additional studies are necessary to establish whether this could be a consequence of a different consumption of fish. Although this fact could reduce human exposure to mercury, a lower consumption of fish and a higher consumption of other meat in the Tapajós region could also contribute to the deleterious long-term cardiovascular consequences. The World Cancer Research Fund International recommends a maximum intake of 500 g of red meat and processed meats per week, because of the high amounts of cholesterol and saturated fat [59]. All of our data point to a possibly easy access to more diversified alternatives for obtaining dietary protein in participants from the Tapajós region, supporting the phenomenon of nutrition transition as proposed by Piperata [7].

Our data also revealed interesting differences between genders in the two regions (Figure 5 and Figure 6). Large differences (more than 10%) were found in the Tapajós region (lower prevalence of female consumers of fruits and male consumers of vegetables) and in the Tucuruí region (higher prevalence of female consumers of eggs and male consumers of meat). 

Taken together, the results of the present study support the need for changes in the eating habits of the populations in both regions, and for an increase in physical activity (not related to housekeeping work) practiced by the female population of the Tapajós region. As mentioned before, individual clinical reports were delivered to each participant with data of anthropometry and eating habits, among others, and with a kit including a measuring tape and a folder explaining how to measure the neck, waist, and hip circumference, the anthropometric limits, and the health problems associated with exceeding these limits. Access to this information and the possibility of performing anthropometric measurements themselves can serve as motivational variables to encourage changes in eating habits and physical activity. However, the design of the tailored behavioral interventions in the two regions can increase the likelihood that changes will occur and continue, improving nutritional status and avoiding the long-term consequences of the worrying results showed by this work. 

### Tailored Behavioral Interventions: Some Possible Nudges Considering Behavior Analysis Principles

We present here a simple set of nudges [12] that incorporates the principles of behavior analysis [13,14], aiming to improve the eating habits of the populations in both regions and increase physical activities practiced by women in the Tapajós region.

Our proposal focuses on favoring the following behavioral changes: (1) to increase fish consumption in the Tapajós region and maintain consumption levels in the Tucurui region and in both region; and (2) in both regions to reduce the consumption of piscivorous fish in favor of the consumption of non-piscivorous fish; and (3) to reduce the daily consumption of cassava flour and rice in favor of fruit and vegetable consumption. The aim of increasing fish consumption in the Tapajos region is based on data that point to the nutritional transition that has been occurring in the Tapajós region with the partial substitution of fish for other meat, considering the cardiovascular risks associated with the increase in meat consumption (e.g., [59]). Secondly, the modification of fish intake according to the type of fish considers the high content of mercury found in piscivorous fish in both regions and the risks of exposure to high levels of this metal [5,26,51]. Furthermore, the change in the intake of cassava flour and rice considers the informal report of many participants on the large quantity of these carbohydrates consumed daily, which may be contributing to the worrying anthropometric data observed in this study. Additionally, in the Tapajós region, the proposal targets favor an increase in physical activities practiced by women.

The implementation of the antecedent component of the nudges can be done through daily reminders on FM/AM radio stations emphasizing the behaviors that should be implemented by the population. These reminders can be recorded by influential people from the Amazon region, such as artists, athletes, religious leaders, politicians, and health agents, among others. Usually, nudges using reminders employ smartphone text messages [60], however, in both regions, this type of device is still hardly used due to the limitations in mobile coverage and electricity supply services. In this context, people usually listen to the radio (battery-operated radio) all day long, and it plays an important role as a source of information and social contact. 

The implementation of the consequent component of the nudges can take advantage of the fact that, in both regions, many participants live in homes with school-aged children. Usually, these families receive economic benefits for keeping children at school (e.g., the Bolsa Família, a government income transfer program mentioned previously), an aspect that can favor family engagement in children’s school activities. This makes it possible to include a weekly record of the anthropometric data explained in the kit distributed to the participants as a part of a child’s school project (Figure 7). The weekly contact with records of the measurements of the neck, waist, and hip circumference, and the information displayed in the folder about the limits and health problems, can act as a consequence for the behaviors we seek to favor: increasing the behaviors recommended in the reminders broadcasted on the radio related to keeping the anthropometric data within limits, and reducing behaviors related to anthropometric data outside the limits. To increase the probability that the records will be accomplished, the record book can be taken by the child to school on a monthly basis to receive an approval stamp from the teacher as a part of a school project. Additionally, this planning allows the carrying out of a natural experiment by comparing the effect of nudges that include contact with the consequences of behaviors (in participants living with children carrying out the school project), and nudges that include only antecedent elements (in participants who do not live with school-aged children or with children who do not carry out the school project).

The effectiveness of the nudges can be evaluated and reviewed every six months, comparing the results of the anthropometric measurements of each participant and their responses on the consumption of different food groups using a similar qualitative questionnaire applied in the present study. The results of the questionnaire can be completed by further comparing the results of the anthropometry, mercury exposure (metal concentration in hair), blood pressure, and glucose levels of each participant obtained previously [10,26] and repeated every six months.

In this context, this study presents the limitation of being a single transversal evaluation that must be confirmed with additional longitudinal studies correlating these anthropometric parameters with the real prevalence of cardiovascular diseases in this population. Furthermore, since the primary data of the nutritional evaluation were registered by the application of a questionnaire, subjective factors can influence the data. Future studies can include follow-ups and direct observers that perform periodic sampling of the food consumption. Despite these limitations, this study provided the first data analyzing the anthropometry-based cardio-metabolic risk in addition to food consumption in Amazonian riverine populations. In this sense, these riverine populations can be considered the “invisible” populations of the Amazon, because they do not have international or institutional visibility (different for indigenous groups), and they hardly appear in the national statistics. Although riverine populations comprise the largest group of traditional populations in the Amazon [7], analyses of risk in these populations are extremely scarce, which eventually contributes to the alarming prevalence of NCDs [10]. Moreover, the interventions proposed here take advantage of the current knowledge of the nutritional and clinical status of these individuals and our experience on the huge limitations to develop efficient strategies in these vulnerable populations. Considering the precarious living conditions and difficult access to healthcare services, tailored behavioral interventions, such as those that we proposed here, would be the best strategies to prevent the deleterious consequences in the near future and to revert the current situation of a public health emergency.

## Figures and Tables

**Table 1:** Limits and classifications of the risk of cardiovascular diseases for diverse parameters used in this study [27,28,29].

BMI (kg/m²)		WC (cm) at CVD Risk	WHR at CVD Risk	NC (cm) at CVD Risk
<8.5	Underweight	≥80 (female)	>0.85 (female)	>34 (female)
18.5–24.9	Normal Weight	≥90 (male)	>1.0 (male)	>37 (male)
25.0–29.9	Pre-obesity			
30.0–34.9	Obesity I			
35.0–39.9	Obesity II			
≥40	Obesity III			

BMI = body mass index; WC = waist circumference; CVD = cardiovascular diseases; WHR = waist-to-hip ratio; NC = neck circumference.

**Table 2:** Anthropometric characteristics of the participants. Data are presented as the prevalence (%) and median (and interquartile ranges) or mean and standard deviation according to the normality of the data. Values above the limits presented in Table 1 are highlighted in bold.

Characteristics		Total	Tapajós		Tucuruí		*P* (Tap vs. Tuc)
			n	%	n	%	
Gender	Male	78	41	29	37	41	0.065 ^a^
	Female	156	102	71	54	59	
	Total	234	143	100	91	100	
Age		44 (31–50)	40 (30–49)		47 (37–51)		0.014 ^b^
Weight (kg)		65.6 (56.6–75.2)	65.6 (56.2–75.0)		65.6 (56.7–76.0)		0.908 ^b^
Height (cm)		157 (151–164)	157 (152–164)		157 (150–166)		0.902 ^b^
BMI		**25.8 (23.0–29.7)**	**25.8 (22.9–29.5)**		**25.5 (23.0–30.1)**		0.992 ^b^
WC (cm)		**90.5 ± 11.9**	**90.3 ± 11.9**		**90.6 ± 11.8**		0.838 ^c^
HC (cm)		101 (96–107)	101 (95–107)		102 (96–107)		0.913 ^b^
WHR		0.90 (0.84–0.94)	0.89 (0.84–0.94)		0.90 (0.84–0.95)		0.855 ^b^
NC (cm)		36 (34–39)	37 (34–39)		36 (34–40)		0.919 ^b^

BMI = body mass index; WC = waist circumference; HC = hip circumference; WHR = waist-to-hip ratio; NC = neck circumference; Tap = Tapajós; Tuc = Tucuruí. ^a^ Fisher’s exact test; ^b^ Mann–Whitney test; ^c^ Student’s *t*-test.

**Table 3:** The prevalence (and number of participants) according to nutritional status (body mass index, BMI) of the riverine populations in the Tucuruí and Tapajós regions. Note: pre-obesity/obesity classification includes pre-obesity, obesity I, II, and III degrees.

Prevalence (and Number of Participants) According to BMI						
Status	Male		Female		Total	
	Tapajós	Tucuruí	Tapajós	Tucuruí	Tapajós	Tucuruí
Underweight	5% (2)	3% (1)	2% (2)	0% (0)	3% (4)	1% (1)
Normal Weight	39% (16)	35% (13)	41% (42)	48% (26)	40% (57)	43% (39)
Pre-obesity/Obesity	56% (23)	62% (23)	57% (58)	52% (28)	57% (82)	56% (51)
Total	100% (41)	100% (37)	100% (102)	100% (54)	100% (143)	100% (91)

**Table 4:** The prevalence (and number of participants) of nutritional status of the pre-obesity/obesity group according to the body mass index (BMI) of the riverine populations from the Tucuruí and Tapajós regions.

Prevalence (and Number of Participants) According to BMI in Pre-Obesity/Obesity						
	Male		Female		Total	
	Tapajós	Tucuruí	Tapajós	Tucuruí	Tapajós	Tucuruí
Pre-Obesity	87% (20)	60.9% (14)	53.4% (31)	50% (14)	63% (52)	54.9% (28)
Obesity I	13% (3)	34.8% (8)	32.8% (19)	35.7% (10)	27.2% (22)	35.3% (18)
Obesity II	0% (0)	0% (0)	12.1% (7)	14.3% (4)	8.6% (7)	7.8% (4)
Obesity III	0% (0)	4.3% (1)	1.7% (1)	0% (0)	1.2% (1)	2% (1)
Total	100% (23)	100% (23)	100% (58)	100% (28)	100% (82)	100% (51)

## Data Availability

All data of this study can be requested to the corresponding authors (ecrespo@ufpa.br; carlosouz@ufpa.br).
